# City-level population projection for China under different pathways from 2010 to 2100

**DOI:** 10.1038/s41597-023-02735-6

**Published:** 2023-11-17

**Authors:** Shangchen Zhang, Mengzhen Zhao, Zhao Liu, Fan Yang, Bo Lu, Zhenping Zhao, Kuiying Gu, Shihui Zhang, Mingyu Lei, Chi Zhang, Can Wang, Wenjia Cai

**Affiliations:** 1 Ministry of Education Ecological Field Station for East Asian Migratory Birds, Institute for Global Change Studies, Department of Earth System Science, Tsinghua University, Beijing, 100084 China; 2 School of Management and Economics, Beijing Institute of Technology, Beijing, 100081 China; 3 School of Linkong Economics and Management, Beijing Institute of Economics and Management, Beijing, 100102 China; 4 Center for Population and Development Studies, Renmin University of China, Beijing, 100872 China; 5 National Climate Center, China Meteorological Administration, NO. 46, Zhongguancun Nandajie, Haidian District, Beijing, China; 6 National Center for Chronic and Noncommunicable Disease Control and Prevention, Chinese Center for Disease Control and Prevention, No. 27 Nanwei Road, Xicheng District, Beijing, 100050 China; 7 Vanke School of Public Health, Tsinghua University, Beijing, 100084 China; 8 State Key Joint Laboratory of Environment Simulation and Pollution Control (SKLESPC), School of Environment, Tsinghua University, Beijing, 100084 China

**Keywords:** Socioeconomic scenarios, Population dynamics

## Abstract

Cities play a fundamental role in policy decision-making processes, necessitating the availability of city-level population projections to better understand future population dynamics and facilitate research across various domains, including urban planning, shrinking cities, GHG emission projections, GDP projections, disaster risk mitigation, and public health risk assessment. However, the current absence of city-level population projections for China is a significant gap in knowledge. Moreover, aggregating grid-level projections to the city level introduces substantial errors of approximately 30%, leading to discrepancies with actual population trends. The unique circumstances of China, characterized by comprehensive poverty reduction, compulsory education policies, and carbon neutrality goals, render scenarios like SSP4(Shared Socioeconomic Pathways) and SSP5 less applicable. To address the aforementioned limitations, this study made three key enhancements, which significantly refines and augments our previous investigation. Firstly, we refined the model, incorporating granular demographic data at the city level. Secondly, we redesigned the migration module to consider both regional and city-level population attractiveness. Lastly, we explored diverse fertility and migration scenarios.

## Background & Summary

Population dynamics profoundly shape economic growth and societal development, influencing consumption and production trends, and correlating with multiple societal issues^1^. For example, the size, composition, and spatial distribution of the population contribute to both emissions and the vulnerability of residents to natural disasters^2,3^. On the other hand, cities are the fundamental units for decision-making processes, which balances the efficiency and relevance of policies^4^. Thus, city-level population data is vital for policy makers in areas of urban planning, shrinking cities, GHG emission projection, GDP projection, disaster risk mitigation, public health risk assessment, etc^5–8^. Though increasing research emphasizes the necessity of high-resolution population forecasts for effective risk management and comprehensive urban planning, city-level population projections for China, one of the most populous countries, are conspicuously absent^9–11^.

In order to address the lack of city-level population projections, an approach that involves aggregating grid-level population projections to the city level can be employed. It is worth noting that grid-level population projections for China already exist. Specifically, a study conducted in 2020 titled “Provincial and Gridded Population Projection for China under Shared Socioeconomic Pathways from 2010 to 2100” employed advanced methodologies, including the consideration of factors such as inverse distance to Population Centre of Gravity, urban fraction, and inverse distance to roads, to downscale provincial-level population data into 1 km grids^12^. In 2022, another study utilized a machine learning method to generate a global population projection at an approximate resolution of 1 km as well^13^. However, it should be acknowledged that there is a deviation of approximately 30% when comparing the actual city-level population trends with the aggregated grid-level projections results in China. This disparity arises due to the existing methods’ inability to adequately capture the underlying dynamics. Firstly, they failed to recognize that city-level population distribution is influenced by various additional factors, including political status, development expectations, and migration policies specific to each city^14^. These factors contribute to the complexity of accurately projecting population trends at the city level. Secondly, they failed to reflect the latest change in total fertility rate (TFR) revealed by the Seventh National Population Census in China. More specifically, after the implementation of fertility policies, the TFR witnessed a substantial increase from 1.50 in 2013 to 1.88 in 2017. However, it subsequently experienced a rapid decline, dropping to 1.30 by 2020, representing the function of fertility policy is far from the expectation. To address these limitations and provide more reliable annual city-level population projections, this study builds upon the cohort-component method used in our previous research in 2020^12^, incorporating three key dimensions of improvement.

Firstly, the study adopts a comprehensive approach by encompassing granular demographic data at the city level, including variables such as sex and age. This enhancement enables a more precise estimation of parameters at the city level.

Secondly, the migration module of the model is redesigned to incorporate the latest policy developments concerning population migration^15^ and accurately depict the varying levels of regional attractiveness, as well as the distinctiveness in attractiveness among cities within a region.

Lastly, a thorough examination of diverse fertility and migration scenarios spanning from 2010 to 2100 is undertaken. This is crucial as the commonly used the SSP (Shared Socioeconomic Pathways), particularly inequality SSP4 and fossil-fuel-dominant SSP5, may be less applicable to China due to recent comprehensive poverty reduction efforts, compulsory education policies, and carbon neutrality goals.

## Methods

As mentioned previously, the present study aims to enhance the existing methodology by incorporating detailed demographic parameters at the city level, revamping the migration module, and subsequently developing a novel set of scenarios. This study establishes 2010 as the reference year, and primarily constructs the input dataset using the data collected during the Sixth National Population Census^16^. Additional data sources utilized for determining parameters are detailed in the section discussing parameter assumptions under different scenarios.

### The projection of city-level population

A recursive multidimensional model is used in this study to project city-level population, including granular details on age and sex. Equations 1a, 2a,b describe a updated version of the recursive multidimensional model, purposefully tailored to accommodate the intricacies of city-level population projection. Specifically, Eq. 1a,b delineate the newborn population of a given city, which is a function of the previous year’s population by age, fertility rates of women at childbearing age, and the neonatal sex ratio.

1a $$P{m}_{yr,a=0}={\sum }_{a=15}^{49}\left(P{f}_{yr-1,a}\times FE{R}_{yr,a}\right)\times B{m}_{yr}$$

1b $$P{f}_{yr,a=0}={\sum }_{a=15}^{49}\left(P{f}_{yr-1,a}\times FE{R}_{yr,a}\right)\times B{f}_{yr}$$

Pm and Pf represent the male and female populations respectively, FER is the fertility rate, and Bm is the ratio of newborn males (Bf for females). The subscript yr is a certain year from 2011 to 2100, a is a certain age from 0 to 99 and a category named “100 and above” (shorted as “100+s”).

Equation 2a and 2b represent the methodologies for iterating the city-level population from one-year-old to those aged “100+“ years.

2a $$P{m}_{yr,a+1}=(P{m}_{yr-1,a}\times (1-MOR{m}_{yr,a})+NetCIMma{d}_{yr,a})\times (1+NetGI{M}_{yr})$$

2b $$P{f}_{yr,a+1}=(P{f}_{yr-1,a}\times (1-MOR{f}_{yr,a})+NetCIMfa{d}_{yr,a})\times (1+NetGI{M}_{yr})$$

MORm is the mortality rate for males (MORf for females), NetCIMmad is the adjusted net city-level migration population for males (NetCIMfad for females), and NetGIM is the net global immigration rate of China.

In this study, Eq. 3a to 3h are employed to delineate migration patterns between cities, while taking into account the latest trends in migration observed between 2011–2020. Given that the Seventh National Population Census in 2020 only provides provincial-level data, this research utilizes a novel methodology to estimate migration characteristics at both the provincial and city-levels. This update significantly enhances the model’s ability to accurately portray the varying levels of regional attractiveness, as well as the distinctiveness in attractiveness among cities within a given region. Specifically, this methodology involves calculating the provincial migration population first, followed by city-level migration population. Additionally, adjustments are made to ensure that the net provincial migration population is equal to 0, and that inter city-level migration population within a province is equal to the migration population of the province.

In Eq. 3a,d, the research presents a detailed explanation of the estimation methodology for both city-level and provincial migration population.

3a $$NetCIM{m}_{yr,a}=P{m}_{yr-1,a}\times NetCIMm{p}_{yr-1.a}$$

3b $$NetCIM{f}_{yr,a}=P{f}_{yr-1,a}\times NetCIMf{p}_{yr-1.a}NetCIMm{p}_{yr-1.a}$$

3c $$NetPIM{m}_{yr,a}=P{m}_{yr-1,a}\times NetPIMm{p}_{yr-1.a}NetCIMm{p}_{yr-1.a}$$

3d $$NetPIM{f}_{yr,a}=P{f}_{yr-1,a}\times NetPIMf{p}_{yr-1.a}NetCIMm{p}_{yr-1.a}$$

NetPIMm is the net provincial migration for males (NetPIMf for females). NetPIMmp is the net provincial migration rate for males (NetPIMfp for females).

It is acknowledged that assumptions regarding provincial and city-level migration rates instead of absolute migration flows can result in imbalances in the migration population, ie. inter-provincial migration population does not equal zero. To this end, the unbalanced excess migration is proportionally cut back in the calculation process. Equation 3e to 3h describe the methodology employed in the calculation process to maintain a balanced migration population.

3e $$0={\sum }_{p=0}^{31}\left(NetPIMma{d}_{yr,a,p}\right)$$

3f $$0={\sum }_{p=0}^{31}\left(NetPIMfa{d}_{yr,a,p}\right)$$

3g $$NetPIM{m}_{yr,a,p}={\sum }_{c=1}^{x}\left(NetCIMma{d}_{yr,a,c}\right)\left({\rm{x}}={\rm{city}}\;{\rm{number}}\;{\rm{of}}\;{\rm{province}}\;{\rm{p}}\right)$$

3h $$NetPIM{f}_{yr,a,p}={\sum }_{c=1}^{x}\left(NetCIMfa{d}_{yr,a,c}\right)\left({\rm{x}}={\rm{city}}\;{\rm{number}}\;{\rm{of}}\;{\rm{province}}\;{\rm{p}}\right)$$

NetPIMmad is the adjusted net provincial migration for males (NetPIMfad for females). NetCIMmad is the adjusted net city-level migration for males (NetCIMfad for females). The subscript p is the province code of a certain province, c is the city code of a certain city within the province p.

### Description of scenarios

To overcome the limitations associated with the application of SSPs in the context of China, we have devised a novel set of scenarios that effectively capture the pivotal factors governing population growth. Population growth is composed of two main components: mechanical growth and natural growth^11,17,18^. Mechanical growth is the change in population resulting from migration, while natural growth is determined by the balance between births and deaths within a population. At the city-level, the migration rate is a critical determinant of mechanical growth, whereas the fertility rate is the key factor influencing natural growth. To facilitate the needs of researchers in selecting the most plausible future scenarios, this study develops a set of five fertility scenarios and three migration scenarios. The detailed descriptions of 15 scenarios are in Table 1. These projections enable researchers to make informed decisions and select the scenarios that are most relevant to their research objectives.

**Table 1:** Demographic assumptions in China under different scenarios.

Scenario	Migration1	Migration22	Migration3	Policies
Fertility1	High Migration Low Fertility	Medium Migration Low Fertility	Stable Migration Low Fertility	Population Ceiling Policy; Reform of Institutional Mechanisms for the Social Mobility of Labor and Talent
Fertility 2	High Migration Stable Fertility	Medium Migration Stable Fertility	Stable Migration Stable Fertility	
Fertility 3	High Migration Medium Fertility	Medium Migration Medium Fertility	Stable Migration Medium Fertility	
Fertility 4	High Migration High Fertility	Medium Migration High Fertility	Stable Migration High Fertility	
Fertility 5	High Migration Extremely High Fertility	Medium Migration Extremely High Fertility	Stable Migration Extremely High Fertility	

### Parameter assumptions under different scenarios

This study presents a set of assumptions regarding mortality, international migration, and sex ratios at birth, which are consistent across various scenarios. Simultaneously, we have designed the fertility and migration components of our analysis to accurately reflect the most recent trends and policies in these domains.

The mortality assumption is based on an expected increase in life expectancy (LE) of one year per decade in each city, taking into account the initial ratio of city-level LE to the national LE in 2010. Using the 2010 census data, the matrix of mortality rates by age, sex, and city is established, resulting in an average growth of 0.96 years per decade in LE in China. This leads to an estimated LE of 87, which aligns with the UN’s medium LE estimation for both genders in 2100^19^.

Regarding international migration, it is assumed that all provinces have the same net immigration rate as the country, which is −0.3015‰ based on the UN’s estimates for 2005–2010 and 2010–2015^19^. This rate remains constant in the second half of the century and gradually declines to zero by 2100.

The sex ratio at birth is assumed to reach 1.07 in 2050 and remain constant thereafter, following the National Population Development Plan’s long-term target^20^.

Regarding fertility, various scenarios are considered and the specific fertility rate assumptions are shown in Table 2. In the low fertility scenario, it is assumed that the fertility policy is insufficient, and the total fertility rate (TFR) drops to 0.7 (the TFR in Shanghai) by 2050^21^, gradually increasing to 0.9 (the TFR in South Korea) by 2100^22^. In the stable fertility scenario, it is assumed that the fertility policy prevents a decline in TFR, but the rate remains constant at 1.3 until 2100. In the medium fertility scenario, it is assumed that the after 2020 fertility policy is sufficient to increase the TFR slowly, reaching 1.5 (the TFR in 2010)^23^ by 2050 and further growing to 1.8 by 2100, the ideal goal of China’s TFR^24^. In the high fertility scenario, the fertility policy is assumed to be effective in promoting TFR, leading to an increase to the ideal value of 1.8 by 2050 and remaining constant thereafter. Finally, the extremely high scenario assumes a successful fertility policy and a change in people’s mindset, resulting in a TFR of 1.8 by 2050 and a replacement level TFR of 2.1 by 2100.

**Table 2:** Assumptions of fertility scenarios.

Target Year	Scenario 1	Scenario 2	Scenario 3	Scenario 4	Scenario 5
2050	0.7	1.3	1.5	1.8	1.8
2100	0.9	1.3	1.8	1.8	2.1

Migration patterns and trends play a significant role in population dynamics. In this study, the migration assumptions consider the expansion of migration scale before 2020, followed by a subsequent decline towards zero by 2035. These assumptions reflect the anticipated changes in migration flows over time.

To analyze provincial migration, the provinces are categorized into two groups based on their respective regions^25,26^. Eastern provinces, with their relatively strong economic status, are expected to exhibit greater attractiveness for migrants. Conversely, middle-western provinces, characterized by slower economic growth, are likely to experience higher negative net migration rates.

Regarding city-level migration, cities are classified into four distinct categories based on several factors including political status, economic strength, city size, regional influence, and population size^27^. These factors collectively depict the developmental status of a city. More developed cities, characterized by advanced medical systems, educational institutions, and robust infrastructure, as well as higher income levels, are expected to exert a greater degree of attractiveness for migrants.

The assumptions underlying the three scenarios in each category are presented in Tables 3, 4, providing a comprehensive overview of the projected population dynamics. In addition to these scenarios, special situations arising from the implementation of the population ceiling policy^28,29^ and the *Reform of Institutional Mechanisms for the Social Mobility of Labor and Talent* policy are also taken into account^15^.

**Table 3:** Assumptions of provincial migration rate in 2020 under different migration scenarios.

Provincial	Scenario 1	Scenario 2	Scenario 3
Middle-West (Loss)	Decrease 50% abNetPIM_p_	Decrease 25% abNetPIM_p_	Same as 2010
East (Gain)	Increase 50% abNetPIM_p_	Increase 25% abNetPIM_p_	Same as 2010

The abNetPIM is the absolute provincial migration rate in 2010. Subscript p is the province code of a certain province.

**Table 4:** Assumptions of city-level migration rate in 2020 under different migration scenarios.

City-level	Scenario 1	Scenario 2	Scenario 3	City List
Tier 1 Cities	Increase 100% abNetCIM_c_	Increase 50% abNetCIM_c_	Same as 2010	Shanghai, Beijing, Guangzhou, Shenzhen
New-Tier 1 Cities	Increase 75% abNetCIM_c_	Increase 25% abNetCIM_c_	Same as 2010	Chengdu, Chongqing, Hangzhou, Xian, Wuhan, Suzhou, Zhengzhou, Nanjing, Tianjin, Changsha, Dongguan, Ningbo, Foshan, Hefei, Qingdao
Tier 2 Cities	Increase 50% abNetCIM_c_	Same as 2010	Same as 2010	Kunming, Shenyang, Jinan, Wuxi, Xiamen, Fuzhou, Wenzhou, Jinhua, Haerbin, Dalian, Guiyang, Nanning, Quanzhou, Shijiazhuang, Changchun, Nanchang, Huizhou, Changzhou, Jiaxing, Xuzhou, Nantong, Taiyuan, Baoding, Zhuhai, Zhongshan, Lanzhou, Linyi, Weifang, Yantai, Shaoxing
Tier 3 Cities and others	Decrease 75% abNetCIM_c_	Decrease 25% abNetCIM_c_	Same as 2010	The rest 313 cities

The abNetCIM is the absolute city-level migration rate in 2010. Subscript c is the city code of a certain city.

Cities designated as megacities with population ceiling policy have implemented restrictions on population size to control their growth by 2035. For instance, Beijing aims to limit its permanent resident population to less than 23 million by adjusting settlement policies. This study assumes that these restrictions are sufficiently effective in preventing further population influx. Therefore, once a city’s population reaches the specified limit, the migration rate is directly set to 0 in that year.

The *Reform of Institutional Mechanisms for the Social Mobility of Labor and Talent* policy seeks to enhance the attractiveness of small cities with populations under 3 million and middle-sized cities with populations ranging from 3 to 5 million by modifying settlement policies. Within this framework, this study assumes that the migration scale of small cities will reduce by 50% before 2020 and 90% thereafter, while the migration scale of middle-sized cities will decrease by 50% before 2020 and 75% thereafter.

## Data Records

The projected yearly city-level, provincial and national population by age, sex, under 15 scenarios for China from 2010 to 2100 are all available at the public repository Figshare^30^ and TsinghuaCloud (https://cloud.tsinghua.edu.cn/f/d593f46793fb4145b8b9/?dl=1).

City-level population data specific demographic attributes for a certain year and scenario are stored in the file “Pop_TOTAL_cityname_SSPFerx_SSPMigrx.csv”, while x represents each scenario number. The file contains two sex groups (M = Male, F = Female), and 101 different age groups (0, 1, 2… 99, 100+). In addition, the total population of each city, the year and the scenario, which are most commonly used in further climate policy studies, are summarized in the file “City_TOTAL.csv”.

Provincial population data specific demographic attributes for a certain year and scenario are stored in the file “Pop_TOTAL_provincename_SSPFerx_SSPMigrx.csv”, while x represents each scenario number. The file contains two sex groups (M = Male, F = Female), and 101 different age groups (0, 1, 2… 99, 100+). In addition, the total population of each province, the year and the scenario, which are most commonly used in further climate policy studies, are summarized in the file “Province_TOTAL.csv”. City name and its corresponding province are summarized in the file “city_name.csv” at the public repository Figshare^30^.

National population data specific demographic attributes for a certain year and scenario are stored in the file “Pop_TOTAL_SSPFerx_SSPMigrx.csv”, while x represents each scenario number. The file contains two sex groups (M = Male, F = Female), and 101 different age groups (0, 1, 2… 99, 100+). In addition, the total population of China, the year and the scenario, which are most commonly used in further climate policy studies, are summarized in the file “Pop_TOTAL.csv”.

## Technical Validation

The projected city-level population is based on the 2010 baseline. Among the various scenarios, the fertility2-migration3 scenario represents stable fertility and stable migration, and can be regarded as the business-as-usual (BAU) scenario commonly used in climate policy research. Therefore, the technical validation primarily focuses on the projection results under this scenario.

To assess the predictive accuracy and bias in the provincial total population and age structure, we employ two widely used accuracy indicators in population projections: the absolute percentage error (APE) and the algebraic percentage error (PE)^12,31^. The equations for these indicators are as follows, where P_t_ represents the projected result and A_t_ represents the corresponding actual value.

4 $$APE\left({\rm{ \% }}\right)=\left|\frac{{P}_{t}-{A}_{t}}{{A}_{t}}\right|\times 100 \% $$

5 $$PE\left( \% \right)=\left(\frac{{P}_{t}-{A}_{t}}{{A}_{t}}\right)\times 100 \% $$

Therefore, projection is overestimated when PE is positive, and vice versa. All actual values that are used for validation are from the latest China National Population Census in 2020^32^.

### Errors in city-level population projection

Table 5 presents the predictive errors observed in the national, provincial, and city-level population projections based on the total population data obtained from the seventh national population census. The national population estimation in our projection is found to be 1.14% larger than the actual value. Regarding provincial projections, the mean APE across all 31 provinces is 3.17%, indicating a slight overestimation with a positive mean PE. Table 6 further illustrates that a majority of provinces exhibit relatively low APEs, with 22 provinces demonstrating APEs below 5% in 2020.

**Table 5:** Errors in the total population projection.

	PE(%)	APE(%)
National	1.14	1.14
Provincial (Mean)	0.82	3.17
City-level (Mean)	3.76	8.43

**Table 6:** Errors of the provincial population projection in 2020.

Province	PE(%)	APE(%)	Province	PE(%)	APE(%)
**Beijing**	−1.40	1.40	**Hubei**	6.24	6.24
**Tianjin**	1.89	1.89	**Hunan**	7.27	7.27
**Hebei**	5.34	5.34	**Guangdong**	−3.27	3.27
**Shanxi**	2.97	2.97	**Guangxi**	6.07	6.07
**Inner Mongolia**	10.49	10.49	**Hainan**	1.69	1.69
**Liaoning**	1.48	1.48	**Chongqing**	−7.43	7.43
**Jilin**	0.80	0.80	**Sichuan**	1.64	1.64
**Heilongjiang**	−0.07	0.07	**Guizhou**	1.77	1.77
**Shanghai**	−0.09	0.09	**Yunnan**	3.00	3.00
**Jiangsu**	5.14	5.14	**Tibet**	−9.27	9.27
**Zhejiang**	−3.07	3.07	**Shaanxi**	0.75	0.75
**Anhui**	−0.12	0.12	**Gansu**	−3.51	3.51
**Fujian**	1.16	1.16	**Qinghai**	−1.46	1.46
**Jiangxi**	−6.69	6.69	**Ningxia**	1.02	1.02
**Shandong**	0.59	0.59	**Xinjiang**	0.96	0.96
**Henan**	1.50	1.50	**Hubei**	6.24	6.24

**Fig. 1:**
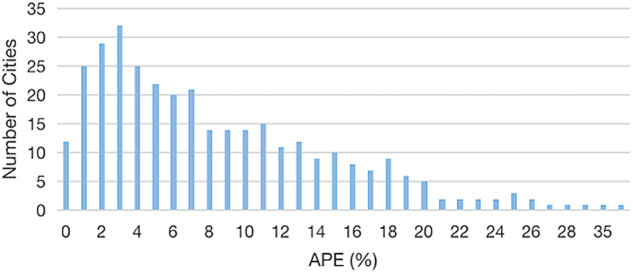
Errors of the city-level population projection in 2020.

To assess the accuracy of city-level population projections, Fig. 1 depicts the distribution of cities across different APE levels. As the administrative boundaries of 24 cities underwent changes between 2010 and 2020, the validation of results is confined to the remaining 338 cities. Notably, 145 cities (42.90%) exhibit an APE of less than 5%, representing 47.05% of the total population. Furthermore, 228 cities (67.46%) have an APE below 10%, accounting for 71.96% of the total population. For the 84 large cities with a population of 5 million or more, the average PE is −0.82, while the average APE stands at 6.95. These error statistics are deemed acceptable in comparison to the errors observed in a county-level population projection for the United States in 2019^33^.

In Table 7, the APEs and PEs are provided for different age groups. Since the seventh population census does not disclose information on provincial 1-year age groups, we utilize the proportion of the population in 15 age groups from the national population census to validate the age information of our projected population. The error results are considered acceptable when compared to the errors observed in the age structure of a provincial population projection for China in 2020^12^.

**Table 7:** Errors in national age structure.

Age Group	PE(%)	APE(%)	Age Group	PE(%)	APE(%)
**0**–**14**	5.44	5.44	**55**–**59**	−0.20	0.20
**15**–**19**	−2.51	2.51	**60**–**64**	0.81	0.81
**20**–**24**	−0.17	0.17	**65**–**69**	−0.61	0.61
**25**–**29**	8.54	8.54	**70**–**74**	0.57	0.57
**30**–**34**	2.36	2.36	**75**–**79**	0.53	0.53
**35**–**39**	1.55	1.55	**80**–**84**	2.30	2.30
**40**–**44**	3.65	3.65	**85+**	6.57	6.57
**50**–**54**	0.55	0.55			

This table shows APEs and PEs in the different population proportion for 15 five-year age groups (0–14, 5–9… 80–84, 85+).

**Fig. 2:**
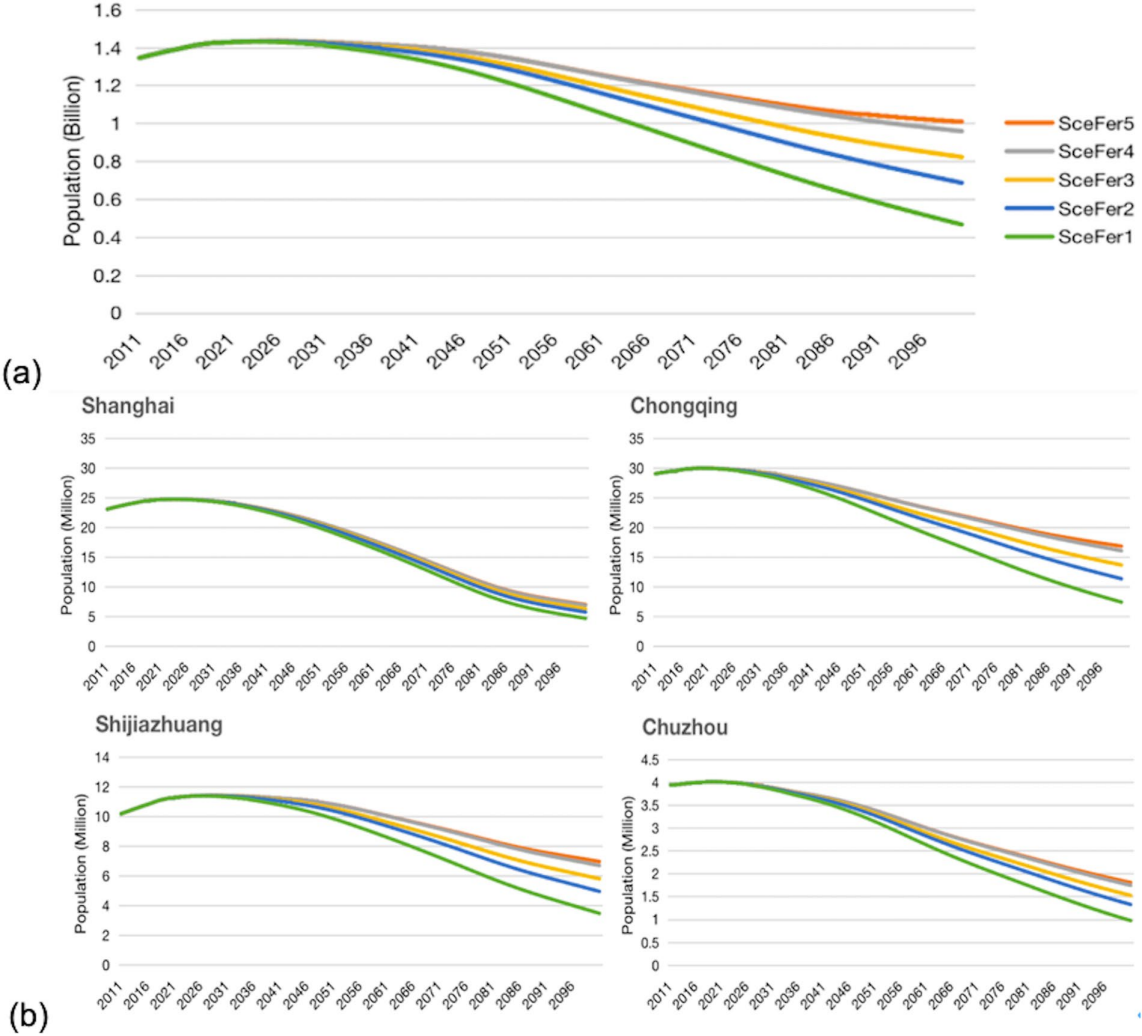
Changes in national, provincial and city-level populations under different fertility scenarios. This figure demonstrates (**a**) future population changes at the national level from 2011 to 2100 under five fertility scenarios, and (**b**) the population changes in sample cities (i.e., Shanghai in Tier1, Chongqing in New-Tier1, Shijiazhuang in Tier2, Chuzhou in Tier3 and others) between 2011 and 2100 under five fertility scenarios.

Figure 2 presents the future trends of the national population and sample city-level populations from 2010 to 2100 across different scenarios. Four sample cities, including Shanghai, Chongqing, Shijiazhuang, and Chuzhou, representing different city categories, are selected for analysis. The national population is projected to peak at 1.43 billion (with a range of 1.43 billion to 1.44 billion) in 2024 (2023–2025). Across all scenarios, the national population demonstrates a significant downward trend. By 2100, the estimated national population ranges from 0.72 to 1.35 billion. It is important to note that different cities exhibit distinct demographic trends due to variations in their initial conditions, developmental capacities, and implemented policies.

## Usage Notes

Scenarios are developed to represent various future development trajectories and mitigate uncertainties by providing a reference range for the key socioeconomic drivers. In our prediction process, we systematically incorporate the influences of multiple factors, including birth, death, and migration, to offer a comprehensive range of future changes in city-level populations. These projections encompass detailed attributes such as sex and age, covering fifteen different scenarios. By considering a broad spectrum of 362 cities in China, including prefecture-level and vice prefecture-level cities, we present a comprehensive set of future population projections at the city, provincial, and national levels under these fifteen scenarios. Researchers can select the desired level and scenario based on their specific research requirements and assumptions regarding future socioeconomic drivers.

However, it is important to acknowledge the presence of uncertainties, particularly those arising from policy-related factors, which can impact both the projections and their distribution. For instance, changes in administrative boundaries for certain cities have been observed, which our model cannot predict. Furthermore, climate-related extremes are anticipated to occur more frequently and with greater severity in the future. As people become increasingly aware of this trend, it is likely to influence the migration patterns of the Chinese population. Unfortunately, our model has not accounted for these factors due to limited data availability. This study is centered on population projections at the city-level, hence we did not break down the city-level population into smaller units. However, we acknowledge that some studies have highlighted the correlation between the distribution of population within a city and the placement of urban facilities, which will support future detailed research based on this study.

This research builds upon the work of Chen *et al*.^12^, but it has been developed and diverges in several significant aspects, making the two studies appropriate for varying research requirements (The core assumptions comparison is placed in the SI). We propose the following guidelines for researchers deciding between the two studies:

- For those requiring projections of populations with different educational levels, the study by Chen *et al*. is most suitable.
- For researchers intending to use population projections for China under SSP4 and SSP5 as extreme scenarios for comparison with scenarios from other countries in global studies, the research by Chen *et al*. would be a recommended option.
- If there is a high demand for the precision of city-level population data, this study is the most appropriate.
- For those wishing to use the most relevant scenarios for China and select the preferred fertility and migration levels based on their needs, this study is the most suitable.
- For researchers needing grid-level population data, a combination of both studies is recommended. Due to the absence of city-level urbanization rates, we do not further downscale our results. However, researchers can utilize the relative population ratios between grids from Chen *et al*.‘s study to downscale our city-level data, and subsequently obtain the desired grid-level data. It is important to note that the relative population ratios between grids only vary among RCPs, hence researchers can select RCP scenarios irrespective of the combined SSPs from Chen *et al*.’s study.

### Supplementary information

Supplementary Information

## Data Availability

All R codes (R3.5.3, https://www.r-project.org) for creating provincial and gridded population datasets for China are stored in public repository Figshare^30^. Explanations are internalized in the script to help users with implementation.
