# Supplementary material for: City-level population projection for China under different pathways from 2010 to 2100
Source: Sci Data. 2023 Nov 17;10:809. doi: 10.1038/s41597-023-02735-6 (PMC10656476; doi:10.1038/s41597-023-02735-6)
Supplement: Supplementary file 1 — Supplementary Information [file 41597_2023_2735_MOESM1_ESM.docx]

**City-level population projection for China under different pathways from 2010 to 2100**

Shangchen Zhang1, Mengzhen Zhao2, Zhao Liu3, Fan Yang4 , Bo Lu5, Zhenping Zhao6, Kuiying Gu7, Shihui Zhang1, Mingyu Lei1, Chi Zhang2, Can Wang8, Wenjia Cai1,*

**Affiliations**

1.Ministry of Education Ecological Field Station for East Asian Migratory Birds, Department of Earth System Science, Tsinghua University, Beijing 100084, China

2.School of Management and Economics, Beijing Institute of Technology, Beijing 100081 China

3.School of Linkong Economics and Management, Beijing Institute of Economics and Management, Beijing,100102, China

4.Center for Population and Development Studies, Renmin University of China, Beijing 100872, China

5.National Climate Center, China Meteorological Administration, NO. 46, Zhongguancun Nandajie, Haidian District, Beijing, China

6.National Center for Chronic and Noncommunicable Disease Control and Prevention, Chinese Center for Disease Control and Prevention, No. 27 Nanwei Road, Xicheng District, Beijing, China 100050

7.Vanke School of Public Health, Tsinghua University, Beijing 100084, China

8.State Key Joint Laboratory of Environment Simulation and Pollution Control (SKLESPC), School of Environment, Tsinghua University, Beijing 100084, China

corresponding author(s): Wenjia Cai (wcai@tsinghua.edu.cn)

**Supplementary Information**

# Core Assumptions Comparison

**Fertility Assumptions in this Study**

| **Target Year** | **Scenario 1** | **Scenario 2** | **Scenario 3** | **Scenario 4** | **Scenario 5** |
| --- | --- | --- | --- | --- | --- |
| **2050** | 0.7 | 1.3 | 1.5 | 1.8 | 1.8 |
| **2100** | 0.9 | 1.3 | 1.8 | 1.8 | 2.1 |

**Fertility Assumptions in Chen et al(2020)**

| **Target Year** | **SSP 1** | **SSP 2** | **SSP 3** | **SSP 4** | **SSP 5** |
| --- | --- | --- | --- | --- | --- |
| **2020** | - | 1.8 | 2 | - | - |
| **2030** | - | 1.65 | - | - | - |
| **2050** | decreases by 25%compared to SSP2 | - | increases by 25%compared to SSP2 | decreases by 25%compared to SSP2 | decreases by 25%compared to SSP2 |
| **2100** | remain constant 2050-2100 | 1.74 | remain constant 2050-2100 | remain constant 2050-2100 | remain constant 2050-2100 |

**Migration Assumptions in this Study**

| **Provincial** | **Scenario 1** | **Scenario 2** | **Scenario 3** |
| --- | --- | --- | --- |
| **Middle-West**  **(Loss)** | Decrease 50% abNetPIM_p_ | Decrease 25% abNetPIM_p_ | Same as 2010 |
| **East**  **(Gain)** | Increase 50% abNetPIM_p_ | Increase 25% abNetPIM_p_ | Same as 2010 |

| **City-level** | **Scenario 1** | **Scenario 2** | **Scenario 3** | **City List** |
| --- | --- | --- | --- | --- |
| **Tier 1 Cities** | Increase 100% abNetCIM_c_ | Increase 50% abNetCIM_c_ | Same as 2010 | Shanghai, Beijing, Guangzhou, Shenzhen |
| **New-Tier 1 Cities** | Increase 75% abNetCIM_c_ | Increase 25% abNetCIM_c_ | Same as 2010 | Chengdu, Chongqing, Hangzhou, Xian, Wuhan, Suzhou, Zhengzhou, Nanjing, Tianjin, Changsha, Dongguan, Ningbo, Foshan, Hefei, Qingdao |
| **Tier 2 Cities** | Increase 50% abNetCIM_c_ | Same as 2010 | Same as 2010 | Kunming, Shenyang, Jinan, Wuxi, Xiamen, Fuzhou, Wenzhou, Jinhua, Haerbin, Dalian, Guiyang, Nanning, Quanzhou, Shijiazhuang, Changchun, Nanchang, Huizhou, Changzhou, Jiaxing, Xuzhou, Nantong, Taiyuan, Baoding, Zhuhai, Zhongshan, Lanzhou, Linyi, Weifang, Yantai, Shaoxing |
| **Tier 3 Cities and others** | Decrease 75% abNetCIM_c_ | Decrease 25% abNetCIM_c_ | Same as 2010 | The rest 313 cities |

**Migration Assumptions in Chen et al(2020)**

| **Income**  **categories** | **Assumptions of migration scenarios** | | | **Province list** |
| --- | --- | --- | --- | --- |
|  | **High** | **Medium** | **Low** |  |
| **High** | Zero NetPIM in 2010 until  2100 | Zero NetPIM in 2020  until 2100 | 50% of current NetPIM in  2030, 0 in 2100 | Beijing, Tianjin, Inner Mongolia, Shanghai,  Jiangsu, Zhejiang, Fujian, Guangdong |
| **Medium** | 50% of current NetPIM in  2100 | 50% of current NetPIM  in 2050, 0 in 2100 | 50% of current NetPIM in  2030, 0 in 2100 | Hebei, Jilin, Heilongjiang, Anhui, Jiangxi,  Shandong, Henan, Hubei, Hunan, Hainan,  Chongqing, Sichuan, Shaanxi, Qinghai,  Ningxia, Xinjiang |
| **Low** | 150% of current NetPIM in  2050 and remain constant | Constant NetPIM | 50% of current 2050 and remain constant | Shanxi, Liaoning, Guangxi, Guizhou, Yunnan, Tibet, Gansu |
